# Postoperative best practice for thumb carpometacarpal joint replacement: a Delphi consensus study

**DOI:** 10.1302/2633-1462.77.BJO-2026-0013.R1

**Published:** 2026-07-01

**Authors:** Nicole Leah Lyons, Shannon Halmkan, Victoria Jansen, Nick A. Johnson, Emma Reay, Ellie Fitzmaurice, Maisie Wilding, Antonina Yakimova, Joy Adamson

**Affiliations:** 1 University Hospitals Tees, James Cook University Hospital, Middlesbrough, UK; 2 York Trials Unit, University of York, York, UK; 3 Pulvertaft Hand Centre, Derby, UK; 4 South Tees NHS Foundation Trust, Middlesbrough, UK

**Keywords:** Osteoarthritis, Delphi analysis, Dual-mobility joint replacement, Thumb carpometacarpal joint, Trapeziometacarpal joint, carpometacarpal joint, hand surgeons, Delphi technique, osteoarthritis, postoperative radiographs, immobilization, splint, osseointegration, radiographs, functional outcomes

## Abstract

**Aims:**

Thumb carpometacarpal joint replacement (CMCJR) is gaining popularity for the surgical management of thumb base osteoarthritis. However, there is considerable variability in the literature regarding postoperative management, with limited information on timescales for return to normal function. This study aims to develop consensus-based guidance for postoperative best practice.

**Methods:**

A three-round modified Delphi process was undertaken with 16 experienced hand surgeons and hand therapists from the UK and mainland Europe. The initial survey was developed following a comprehensive review of the literature and consultation with a patient and public involvement group. The second round was an iteration of the first, including refinement of themes into statements. The third round presented the guidance for overall agreement and comment. Consensus throughout was defined as ≥ 70% agreement.

**Results:**

In round one, consensus was reached on postoperative immediate protection duration of ten to 14 days and the need for a postoperative radiograph. In round two, agreement was achieved for seven out of ten statements that included the type of immediate postoperative protection to be a bulky bandage allowing restricted thumb movement, splinting practices, thumb full range of motion commencement, and a return to driving timescale of four weeks. Return to timescales of light (two weeks), moderate (four weeks), and heavy tasks (six to eight weeks) were further defined in round three and accepted by the panellists.

**Conclusion:**

This Delphi study established consensus-based guidance for best practice in the postoperative management of thumb CMCJR, with specific inclusion of timescales for return to normal function. This will be beneficial for clinics, particularly in the UK, which are newly adopting this technique. Recommendations regarding the timing and frequency of postoperative radiographs remain to be clearly defined.

Cite this article: *Bone Jt Open* 2026;7(7):825–837.

## Introduction

Thumb base osteoarthritis (TBOA) is one of the most common locations for OA in the hand, and while there are many known risk factors, age and sex are the most well known.^1-3^ The functional impact of painful TBOA can negatively affect activity performance, work ability, and health-related quality of life.^4^ It is estimated to contribute to work absenteeism in up to 16% of employed individuals.^5^

It is generally accepted that surgical intervention for the management of TBOA should be considered if staged non-surgical treatment has been ineffective.^6,7^ Trapeziectomy is the most commonly performed surgical procedure for TBOA. However, as of 2020, it was estimated that approximately 13% of European hand surgeons preferred thumb carpometacarpal joint replacement (CMCJR) over trapeziectomy, with a recent trend towards the use of dual-mobility implants.^8^

While thumb CMCJR is on the increase, studies on dual-mobility prostheses reveal considerable inconsistency in postoperative management. Notably, the duration of postoperative immobilization varies widely, ranging from no immobilization,^9,10^ to periods of one week,^11-14^ three weeks,^15-18^ or six weeks.^19^ Authors’ preferences on how to immobilize the thumb postoperatively also differ, from the application of a bulky bandage to rigid casting.^11,13,20,21^ Additionally, some advocate for the subsequent use of an orthosis following the initial period of immobilization.^11,13,14,21,22^ Hand therapy is often not mentioned or clearly described in the literature, and some advocate for patient self-directed rehabilitation with no restrictions, reserving referral to hand therapy only in cases where patients exhibit difficulties in functional progression.^15,23^ Furthermore, the latest generation of dual-mobility prostheses available in the UK do not come with clear guidelines for rehabilitation, and suggest three to four weeks of immobilization, or surgeon’s preference.^24,25^

A further scoping review concluded there is no scientific evidence for the superiority of any postoperative regime following thumb CMCJR.^26^ No conclusions can be drawn about the effects of early active mobilization, or complications specifically related to a given immobilization or rehabilitation protocol. In the absence of a robust evidence base to provide postoperative recommendations, we employed a modified Delphi approach to establish consensus-based guidance for postoperative care following thumb CMCJR.

## Methods

**Fig. 1:**
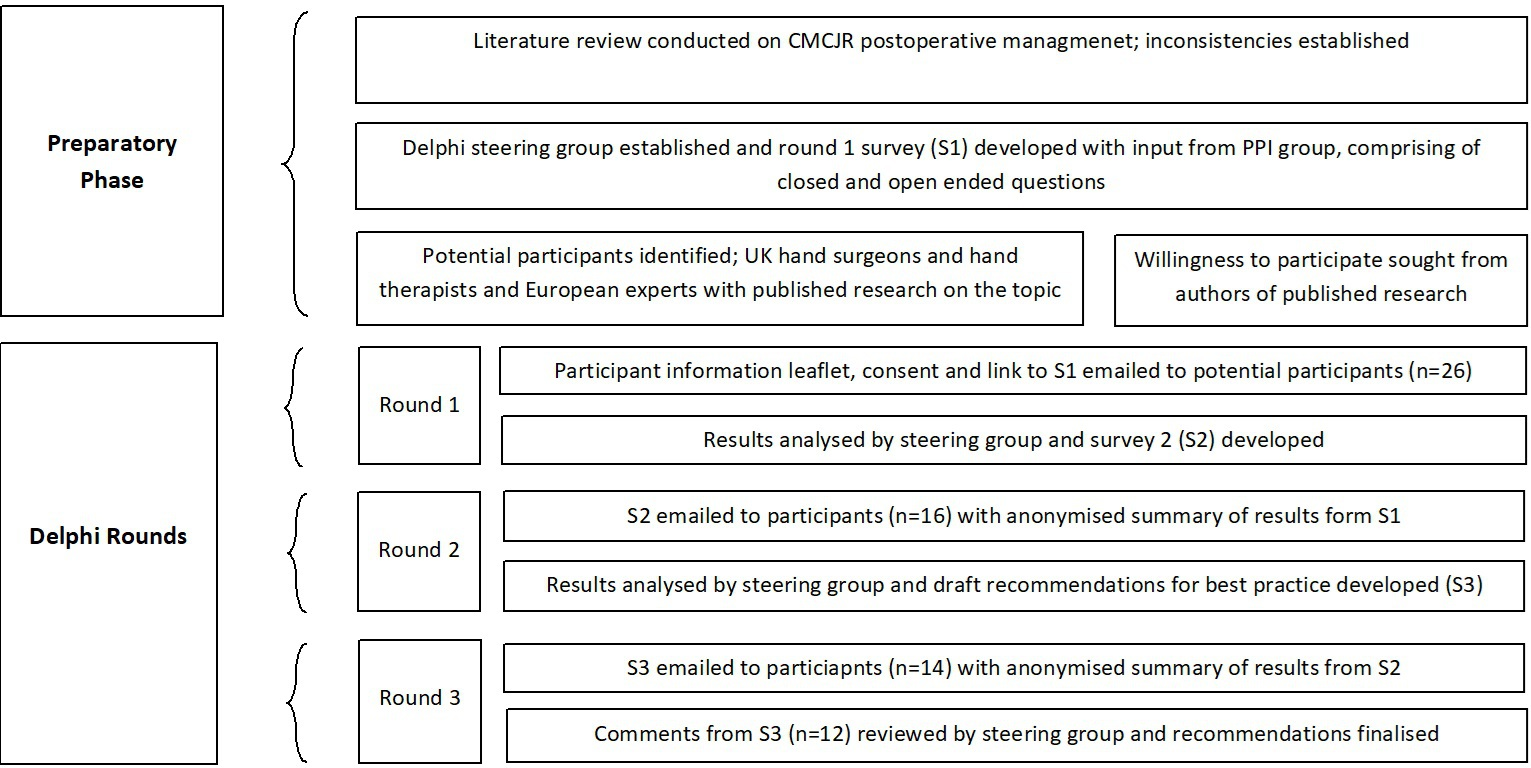
Three-round modified Delphi process. CMCJR, carpometacarpal joint replacement; PPI, patient and public involvement.

The aim of the Delphi method is to provide a group facilitation technique, designed to transform opinion into group consensus through anonymous, iterative rounds of surveys with controlled feedback and statistical analysis.^27^ The process can be successfully adopted in health-related research to address areas of uncertainty where empirical evidence is lacking or in conflict,^28^ as is the case here. Drawing upon the established methodological framework for health-focused Delphi studies,^29^ this research implements a three-round modified Delphi method to investigate and harmonize variations in postoperative management protocols for thumb CMCJR, which is summarized in Figure 1.

### Ethics

Ethical approval for this study was obtained from the University of York, Department of Health Sciences Research Ethic Committee on 20 May 2024.

### Survey development

A Delphi steering group was formed to investigate consensus, consisting of two hand therapists, two hand surgeons, three health researchers, and one statistician. The role of the steering group was to provide guidance around the content development of the Delphi questionnaires and deliver feedback on the preliminary analysis of results. The initial round one survey was developed following a review of the literature and input from a patient and public involvement (PPI) group, comprising six patients who had undergone surgical treatment for TBOA. The PPI group strongly conveyed the importance of timescales for returning to certain functional activities and definitions of light, moderate, and heavy tasks. Consequently, three themes were identified to explore; immediate postoperative phase, mobilization phase, and return to normal function. Composition of the survey included a mixture of closed and open-ended questions to give panellists the opportunity to put forward their opinions. Demographic and information determining level of experience was included. The online secure survey design platform Qualtrics was used to facilitate ease of participation from a geographically diverse panel. Panellists were approached to take part in the study via email, which detailed the study information, commitment expectations, privacy notice, and a link to the survey if electronic informed consent was provided by the panellists, all of which is available to view in the Supplementary Material.

In rounds two and three, panellists were presented with anonymized summary results from the previous round, in keeping with the Delphi process of allowing panellists the opportunity to reflect on and reconsider any points.^30^ Panellists were blinded to individual responses. The second-round survey was an iteration of the first with open-ended questions now formed into statements where possible, seeking agreement on acceptability to develop postoperative recommendations. Where uncertainty still existed, the topics were further explored with controlled feedback. In the final round, participants were presented with the draft postoperative consensus-based guidance and provided with the opportunity to add any comments on the acceptability of these.

### Consensus

Generally, up to a nine-point rating scale is recommended for a consensus process, with a mean rating of seven or above indicating statement agreement.^31^ However, a smaller scale seems to yield improvements in consensus over a five- or nine-point scale,^32^ and this study used a two-point rating scale (agree/disagree), to maximize clarity of statement consensus. While there is no clear consensus on what the threshold percentage should be for Delphi studies, it is suggested that less than 70% would affect study validity,^33^ therefore a consensus threshold of 70% was chosen.

### Eligibility criteria and recruitment

Two stakeholder groups across the UK and Europe were targeted for recruitment: 1) specialist hand surgeons performing CMCJR; and 2) hand therapists who had considerable experience in postoperative management of CMCJR. Panellists were identified through publication records, conference presentations, and snowball sampling. As it was likely that practice would be similar between clinicians within sites, surgeons and hand therapists from the same site were not included.

**Fig. 2:**
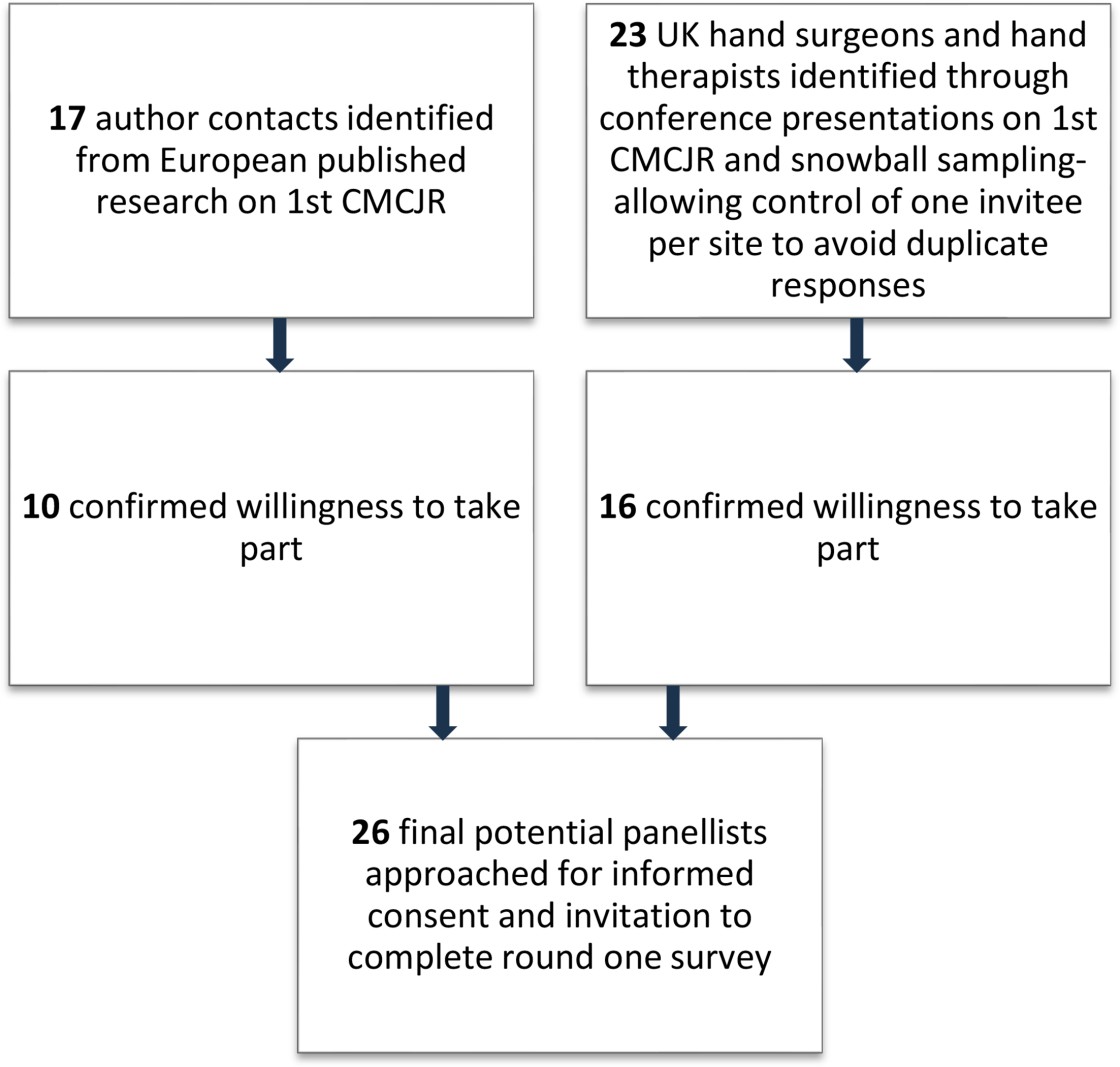
Recruitment process for round one. CMCJR, carpometacarpal joint replacement.

Willingness to take part was sought prior to the invitation email, which led to the identification of 26 potential panellists (Figure 2). Significant variation exists in published studies around Delphi sample sizes, with recognition that larger sample sizes can improve reliability of findings, hence a proposed minimum of eight to ten is often recommended.^34-36^ A total of 26 potential panellists ensured the recommended minimum sample size was achievable, while accounting for predictable attrition between rounds.^37^ To enhance stakeholder engagement with the Delphi process, participants were offered voluntary entry into a free prize draw and study contributor acknowledgement upon completion of all rounds.

### Statistical analysis

Descriptive statistics were used to analyze the responses, providing frequencies and percentages on all items after each round to identify any that reached the consensus threshold of ≥ 70%. Free-text responses were discussed with the steering group between rounds, with care taken to remove any participant identifiable information, respecting anonymity. Study reporting followed the Accurate Consensus Reporting Document (ACCORD) guideline.^38^

## Results

**Fig. 3:**
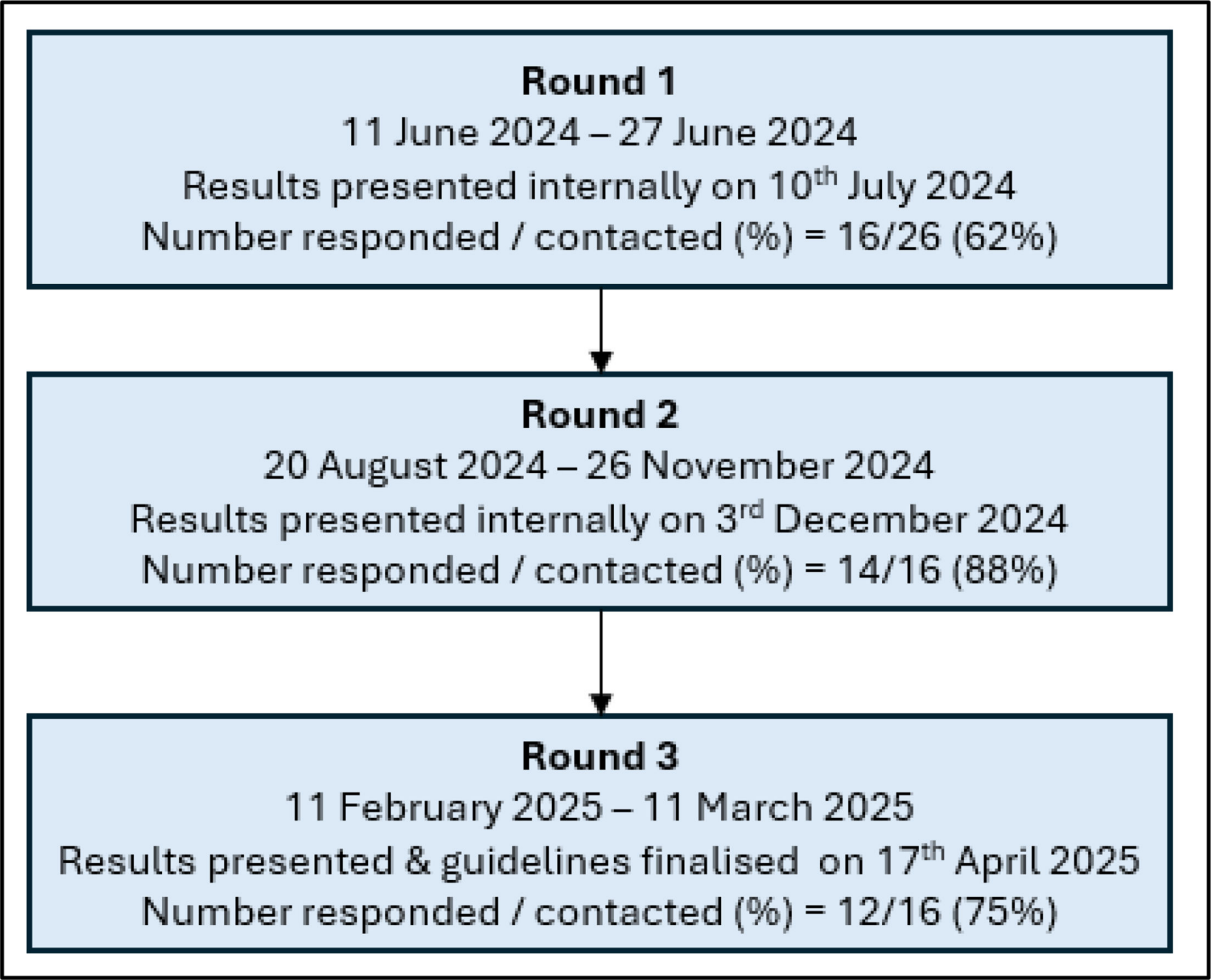
Flowchart to highlight dates of data collection and data analysis for rounds one to three. Response periods were increased in rounds two and three from the proposed two weeks to encourage a higher completion rate.

Data collection for the Delphi study took place from 27 June 2024 to 11 March 2025. In total, 26 specialists were contacted, among whom one advised they were ineligible and 16 (62%) completed the first round. Out of the initial 16 responders, 14 (88%) and 12 (75%) also responded to rounds two and three respectively; Figure 3 highlights the data collection and analysis period for each round. Full demographic details and details of experience in CMCJAs are outlined in Table I, which demonstrates broad geographical distribution and evidence of advanced experience of the panellists.

**Table I.** Demographic details of survey respondents for each round.

Variable, n (%)	Round 1 (n = 16)	Round 2 (n = 14)	Round 3 (n = 12)
**Sex**			
Male	9 (56)	8 (57)	8 (67)
Female	7 (44)	6 (43)	4 (33)
**Age group, yrs**			
30 to 49	11 (69)	9 (64)	7 (58)
50 to 59	5 (31)	5 (36)	5 (42)
**Location**			
Continental Europe	6 (38)	5 (36)	4 (33)
UK	10 (62)	9 (64)	8 (67)
**Occupation**			
Hand surgeon	11 (69)	9 (64)	8 (67)
Hand therapist	5 (31)	5 (36)	4 (33)
**Type of clinic**			
NHS	9 (56)	8 (57)	7 (58)
Other*	7 (44)	6 (43)	5 (42)
**Experience using CMCJR for TBOA, yrs**			
≤ 10	8 (50)	6 (43)	5 (42)
> 10	8 (50)	8 (57)	7 (58)
**Applicable to surgeons only**			
**Average number of CMCJRs per annum**			
≤ 30	6 (55)	4 (44)	4 (50)
> 30	5 (45)	5 (56)	4 (50)
**Most commonly used implants**			
TOUCH (KeriMedical, Switzerland)	8 (73)	6 (67)	5 (62)
MAIA (Groupe Lépine, France)	3 (27)	3 (33)	3 (38)

* Other clinic types included: private healthcare, European public healthcare, university hospital, NHS, and private combination.

CMCJR, carpometacarpal joint replacement; TBOA, thumb base osteoarthritis.

### Round one

Of the 16 panellists in the first round, 11 were surgeons and five were hand therapists. It should be noted that one panellist provided only demographic data in round one; the decision was made not to exclude them from analysis since they participated fully in rounds two and three. The full survey given to panellists in round one is provided in the Supplementary Material.

**Fig. 4:**
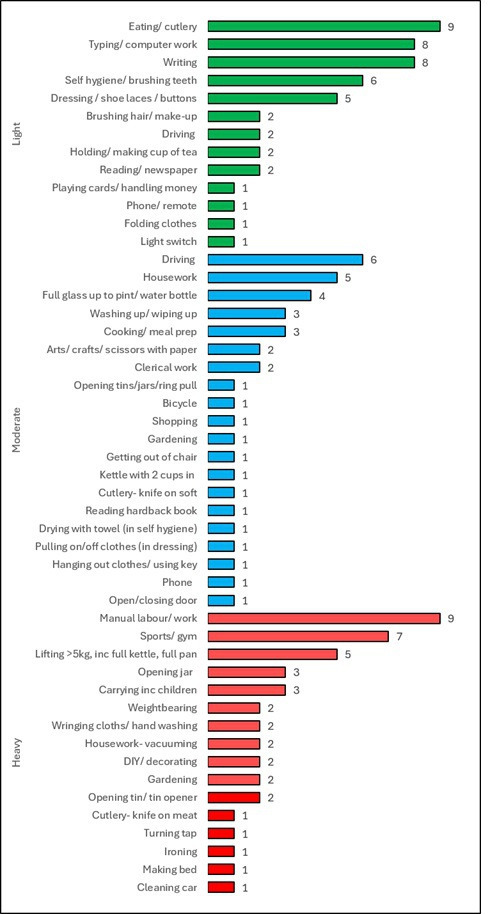
Bar chart to highlight the tasks identified by panellists in round one.

Responses to the first round are summarized in Table II. The consensus threshold of ≥ 70% was reached for two items. In particular, the length of postoperative immobilization to be ten to 14 days and agreement with the need for postoperative radiographs; these items were removed for round two. Panellists were asked to give examples of light/moderate/heavy tasks when considering returning to normal function. Further details of the examples given are provided in Figure 4.

**Table II.** Round 1 item agreement (percentages relative to n = 15 participants providing data).

Item	N agreed (%)*
**Immediate postoperative phase**	
**Preferable duration of postoperative immobilization**	
10 to 14 days	11 (73)
5 to 7 days	3 (20)
From day 1	1 (7)
**Preferable immediate postoperative support**	
Bulky bandage maintaining first webspace	7 (47)
Bulky bandage with hard plaster component maintaining first webspace	4 (27)
Thumb spica plaster cast	3 (20)
Ready-made orthosis soft	1 (7)
**Mobilization phase**	
**Use of additional thumb splinting after immobilization**	
No	1 (7)
Only if required	4 (27)
Always	10 (67)
**Type of thumb splint offered (used always, n = 10)**	
Ready-made rigid splint	1 (10)
Ready-made soft splint	4 (40)
Thermoplastic fabricated splint	4 (40)
Other†	1 (10)
**Type of thumb splint (used only if required, n = 4)**	
Ready-made rigid splint	3 (75)
Ready-made soft splint	1 (25)
**Timescale of thumb splint weaning off**	
4 to 6 weeks (4 not inclusive)	6 (40)
3 to 4 weeks	5 (33)
1 to 2 weeks	3 (20)
Missing	1 (7)
**Approach to postoperative rehabilitation**	
Patient is only referred to hand therapy if required	1 (7)
Patient is referred to hand therapy for postoperative rehabilitation	9 (60)
Patient self-manages with an advice and exercise leaflet	3 (20)
Patient education and advice, no exercises	1 (7)
Specialist nurse	1 (7)
**Commencement of thumb exercises**	
Immediately	3 (20)
After 1 week	2 (13)
After 2 weeks	10 (67)
**Timescale to return to normal function**	
**Return to light tasks**	
Immediately	3 (20)
1 to 2 weeks	9 (60)
3 to 4 weeks	3 (20)
**Return to moderate tasks**	
1 to 2 weeks	2 (13)
3 to 4 weeks	6 (40)
5 to 6 weeks	6 (40)
Missing	1 (7)
**Return to heavy tasks**	
5 to 6 weeks	3 (20)
7 to 8 weeks	8 (53)
9 to 10 weeks	1 (7)
Greater than 10 weeks	3 (20)
**Return to driving**	
1 to 2 weeks	2 (13)
3 to 4 weeks	9 (60)
5 to 6 weeks	3 (20)
7 to 8 weeks	1 (7)
**Postoperative radiograph**	
**Need for postoperative radiographs**	
No (unless there is a problem)	1 (7)
Yes	14 (93)
**Intervals for postoperative radiographs (answers are not mutually exclusive)**	
1 to 2 weeks	8 (53)
3 to 4 weeks	1 (7)
5 to 6 weeks	8 (53)
Greater than 6 weeks	5 (33)

* Percentages may not round exactly to 100% due to the laws of rounding.

† Other option given was: ‘[soft] in daytime then rigid at night’.

### Round two

**Fig. 5:**
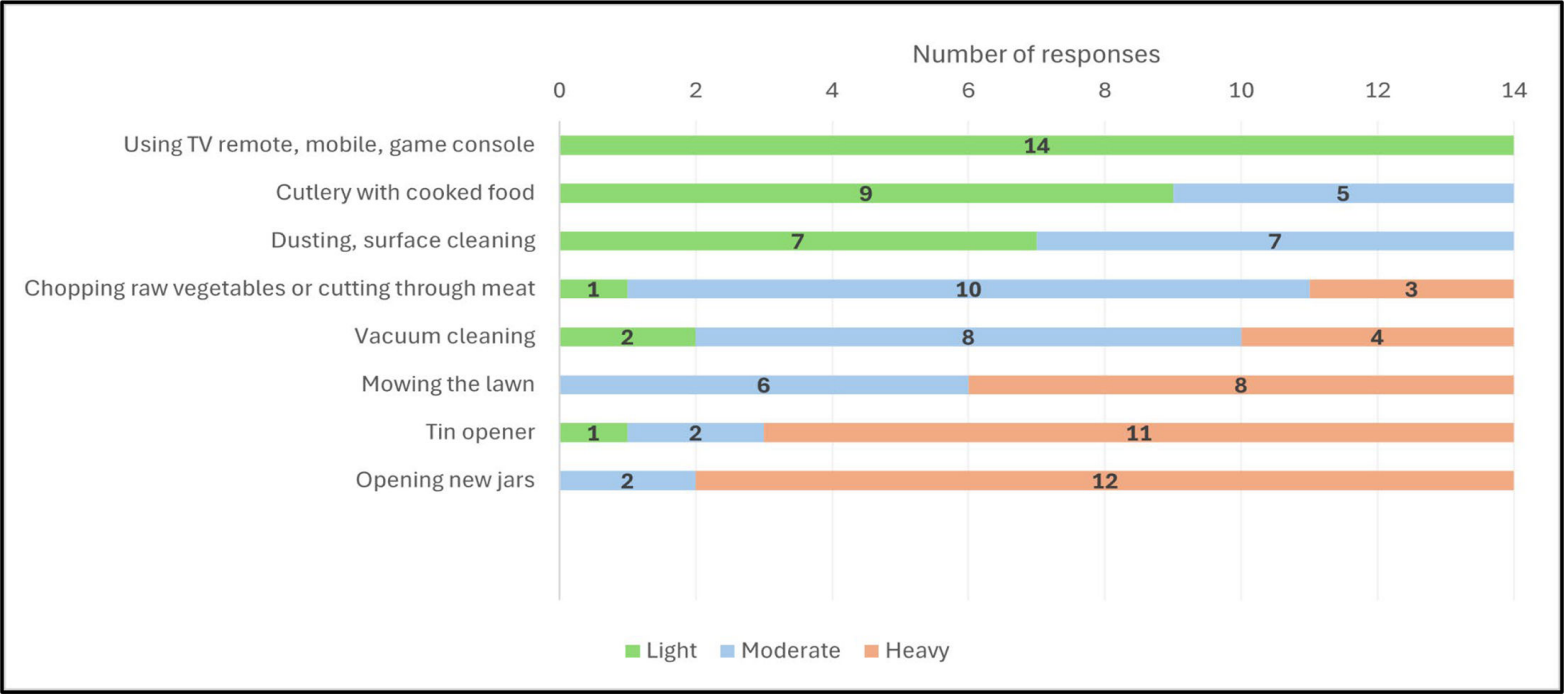
A stacked bar group to show tasks classified on severity by round 2 participants.

The 14 respondents to round two consisted of nine surgeons and five hand therapists. The consensus level of ≥ 70% was achieved for seven out of the ten (70%) given statements in this round. Levels of agreement are summarized in Table III. The remaining disagreement consisted of: the necessity of supervised postoperative hand therapy sessions, the advisement of a splint when driving, and the need for further radiographs when there are no clinical concerns. Timing of radiographs and commencement of postoperative tasks (by difficulty) were also explored but did not reach a consensus. The minimum timeframe with a cumulative agreement level of at least 70% was used to determine round three draft guidance. Further refinement on categorizing tasks by difficulty when returning to normal function was obtained and summarized in Figure 5; the consensus threshold was reached for 4/8 (50%) of the listed examples. Where consensus was not achieved, a task was categorized with the higher level of difficulty to be conservative.

**Table III.** Agreement levels of items in round 2 (percentages relative to n = 14 participants completing this round)

Statement	Agreement with statement, n (%)
**Immediate postoperative phase**	
For the majority of uncomplicated cases, a bulky bandage maintaining first webspace (which could allow for some restricted range of motion) provides enough support postoperatively	13 (93)
**Mobilization phase**	
After the initial period of immobilization, for the majority of uncomplicated patients, intermittent daytime splinting can be offered as required for pain relief and protection according to individual lifestyle needs	14 (100)
After the initial period of immobilization, for the majority of uncomplicated patients, night-time splinting is **not** necessary	11 (79)
**Weaning from additional support**	
For those offering an additional splint, we would suggest that this additional support can be gradually reduced within the 6-week postoperative period	14 (100)
**Postoperative movement, exercise, and rehab**	
For the majority of uncomplicated patients, would you be happy with self-management beginning after one postoperative visit, when the patient has been provided with education and advice and an additional support if necessary, following the initial postoperative period of immobilization?	8 (57)
**Commencing thumb active range of motion**	
In a straightforward patient, it is safe to allow some active range of motion within the confines of a bulky postoperative dressing (with no hard component)	14 (100)
In a straightforward patient, it is safe to allow full active range of motion once the initial postoperative dressing/support has been removed	14 (100)
**Return to normal function**	
For the majority of uncomplicated patients, return to driving can be recommended within 4 weeks if they are comfortable to do so	10 (71)
It is not necessary to advise a splint while driving before 6 weeks post-operation	9 (64)
**Recommended starting time of tasks (by difficulty)**	
**Light tasks**	
Immediately	2 (14)
From 1 week	5 (36)
From 2 weeks	7 (50)
**Moderate tasks**	
From 2 weeks	2 (14)
From 3 weeks	5 (36)
From 4 weeks	5 (36)
From 6 weeks	2 (14)
**Heavy**	
From 6 weeks	7 (50)
From 7 weeks	2 (14)
From 8 weeks	4 (29)
From 10 weeks	1 (7)
**Postoperative radiograph**	
**Timescale for the first radiograph**	
1 to 2 weeks/first review	5 (36)
4 weeks	1 (7)
At least 6 weeks	7 (50)
If there are no clinical concerns, there is no need to repeat radiographs after the initial postoperative radiograph	8 (57)

### Round three

**Fig. 6:**
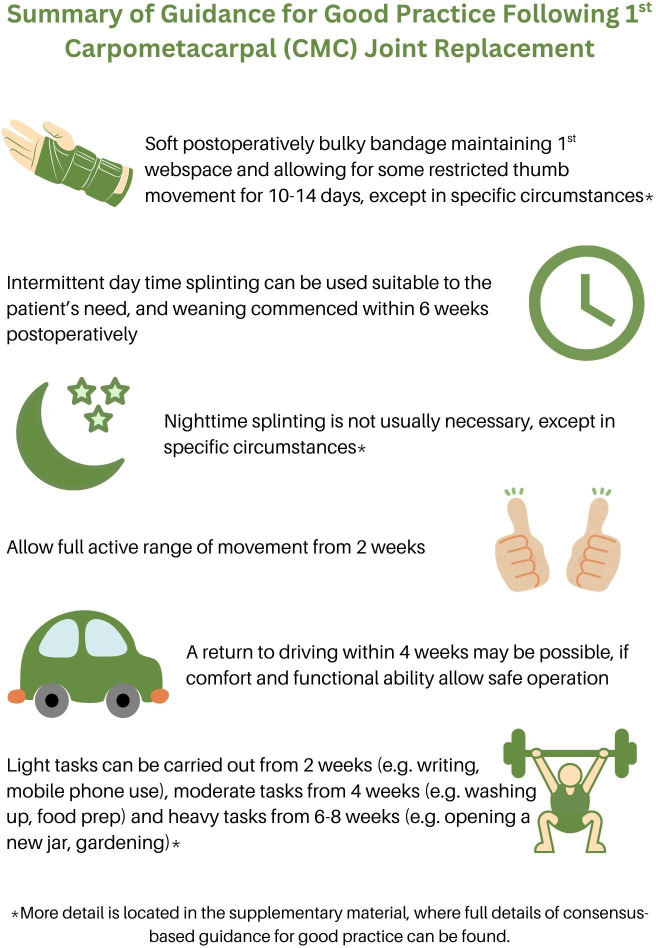
Infographic highlighting the main aspects of the finalized recommendations.

Based on agreement in rounds one and two, the consensus-based guidance was proposed in round three. The 12 panellists who responded to round three (eight surgeons, four therapists) indicated agreement with the guidance either explicitly or by suggesting no further modifications; therefore, the proposed guidance was considered final. An infographic is provided in Figure 6 highlighting the main aspects of the guidance; the full version (Supplementary Material) provides more advanced detail on return to function.

## Discussion

A modified Delphi technique has been used to create consensus-based guidance for the postoperative management of thumb CMCJR. The guidance is intended for uncomplicated postoperative patients, who have a predictable recovery trajectory defined by manageable pain, ability to achieve early movement, and the absence of intraoperative complications (e.g. bone strength issues including trapezium fracture) or acute postoperative complications (e.g. infection, tendon rupture, and prosthesis dislocation).

There was consensus on providing ten to 14 days of postoperative protection with a soft bulky bandage for uncomplicated cases, which allows for early controlled thumb range of motion (ROM). There remains a lack of clinical research directly comparing immobilization times with functional outcome. However, there is evidence to suggest low complication rates and favourable functional outcomes with minimal immobilization.^9,10,12,14^ This is reflected in established practices in high-volume centres across Europe, whereby one panellist observed immobilization beyond one week is unusual. In contrast, consistent improvements in ROM and function have also been reported with dual-mobility prostheses immobilized for three weeks postoperatively.^16-18^ Heterogeneity in outcome measures between these studies do not allow for accurate comparison to advocate the optimum immobilization time. Here, the consensus among panellists of this study was that restricting mobility for up to two weeks did not hinder recovery of thumb ROM and allowed time for soft-tissue structures to settle. This area warrants ongoing monitoring and additional research to support the development of future evidence-based recommendation.

Effective osseointegration is integral to the success of thumb CMCJR, and early controlled movement and mechanical loading are posited to stimulate osteogenesis. Indeed, the design of the dual-mobility thumb CMCJR is similar to hip prosthesis, where studies highlight the safety of day zero movement and ambulation with subsequent faster return to function compared with constrained cups, with no evidence of increased osseointegration failure or increased rate of revision surgery.^39-41^ Although these data cannot be directly transferrable to CMCJR, it is nonetheless encouraging. Concern regarding osseointegration failure was expressed by a low number of panellists in this study, contributing to a more conservative immediate postoperative approach and return to function in round one. Clinically, failure of osseointegration typically manifests as early implant loosening. Notably, the use of dual-mobility prosthesis appears to mitigate this risk, with studies reporting no evidence of early loosening and low rates of late loosening (ranging from 0% to 2%) in follow-ups exceeding three years.^17,23,42^

The use of a splint following the initial postoperative immobilization period appears to vary across clinical practice (Table II). While all hand therapists expressed support for splint use, surgeons demonstrated a broader range of opinions and confessed that choice was often limited to what was available in the clinic. Studies reporting splint use vary in type and wearing regimes, with no specific justification provided for use.^11,13,14,43,44^ Here, panellists did provide rich informative feedback with regard to rationale, and often emphasized the need for flexible, patient-specific decision-making. Panellists mentioned adjusting decisions based on pain, lifestyle demand, soft-tissue tightness, and the risk of overuse. This included tailoring splint type – from soft neoprene wraps to bespoke thermoplastic splints – to accommodate factors such as early support during function, facilitating rest/comfort, behaviour cues to mitigate overuse, or correcting significant soft-tissue tightness. Panellists also mentioned that weaning should take into consideration pain, stability, and functional independence, with observations that most patients are comfortable without a splint by four to six weeks. Equally, several panellists expressed that not all patients required routine splinting, highlighting the variability in recovery trajectories. Similar observations are reported in studies examining splinting practices following surgical trapeziectomy for TBOA, where it is theorized that the level of support provided should be determined according to the individual patient’s needs.^45^

To date, no study has systematically categorized specific activities into light, moderate, or heavy tasks, and few have reported detailed timelines for return to activities of daily living (ADL), including driving. In this study, the panellists predictably suggested low-loading non-repetitive tasks, quantified as light, reserving power grip and forced pinch activities for the heavy category. Moderate activity seemed more difficult to quantify, but panellists agreed that most ADL, outside of self-care (light), belonged in this category. While biomechanical studies have investigated load transmission across the native thumb joint during ADL, there is currently no clinical literature evaluating load transmission through a thumb CMCJR under functional loading conditions.^46,47^ However, the limited number of studies reporting mean time to return to function, together with low rates of complications such as dislocation and implant loosening, provide indirect support for the timescales adopted in the present study. Moscato et al^9^ referenced a mean return time of 21.6 days for routine ADLs, aligning with the current study’s guidance to resume moderate activity within four weeks. Furthermore, previously reported mean return-to-work (including manual labour) intervals of 44 and 48 days provide additional support for this study’s guidance advocating a return to heavier functional use between six and eight weeks.^10,22^

In this study, a greater proportion of more experienced surgeons (> five years) considered supervised rehabilitation unnecessary to regain function. The only comparative study in the literature examining supervised compared with unsupervised hand rehabilitation following thumb CMCJR found no significant or clinically relevant advantage of supervised rehabilitation.^48^ It is accepted in this study that the delivery of education and support will vary in format, but formally prescribed set amounts of supervised sessions do not seem necessary; instead, a programme tailored to the patient is recommended.

No consensus on best practice for postoperative radiograph frequency was possible, with clear division in opinions on whether more than one radiograph was required. Recent evidence suggests that routine postoperative radiographs following un complicated hip and knee arthroplasty may not be necessary within the first one to ten years after surgery.^49^ These imaging practices, once thought essential for early detection of complications, have shown limited clinical value in asymptomatic patients, and may contribute to unnecessary healthcare costs and radiation exposure.^50^ Current UK guidelines for major joint arthroplasties now recommend that follow-up radiographs be reserved for cases where new or unexplained symptoms arise, however as thumb CMCJR implants are relatively new with limited follow-up data, some radiological surveillance seems sensible.^51^

This consensus project provides guidelines based predominantly on the practice of very experienced UK and European CMCJR surgeons. Many of the surgeons in the UK are not as experienced in the practice of CMCJR, and the consensus-based guidance may have the benefit of providing a framework of acceptability for postoperative protocols. The disadvantage of having such experienced surgeons informing the Delphi process is that their recommendations may not be accepted by less experienced surgeons, who are often more comfortable immobilizing patients for longer and providing more postoperative rehab.^52^

There are several areas where further research will be needed to help support the postoperative guidance made in this paper. Further high-level evidence is needed regarding the method and duration of immobilization, and the intensity and duration of postoperative rehabilitation. The use of postoperative imaging is also an area which will need more in-depth research to allow longer-term surveillance of the CMCJR implants as they are undertaken more widely in the NHS to bring them in line with other, more established implants.

A total of 26 participants were invited to take part in round one, with 16 responding and 12 completing all three rounds. Although there is evidence to suggest that this is an adequate number for a Delphi study,^35^ the small number of panellists may result in the findings not being generalizable, and so could be a limitation of this study. Furthermore, 10/16 (63%) of the responders in round one were UK-based, which reflects a greater bias towards what is happening in UK practice and may reduce the applicability of the guidance more internationally. It is also noted the two stakeholder groups were unequally matched, with 11/16 (69%) participants in round one comprising of hand surgeons. However, the only obvious difference between these two stakeholder groups were their opinions on supervised compared with unsupervised rehabilitation, with the hand therapists leaning significantly more towards supervised. Lastly, in the third and final round, the only way a participant could comment on the recommended guidelines was via the following free-text question: “If you have any feedback regarding the guidelines, please comment below*”.* Although all 12 panellists reviewed the guidance, only 5/12 (42%) provided a response to this question. In all five cases, agreement was expressed. Since a response was not required, the remaining seven did not provide any further feedback or modifications. Based on how the question was phrased, it was considered sensible to assume they agreed with the suggested guidance.

In conclusion, there is a notable paucity of research directly comparing postoperative protocols following thumb CMCJR, and considerable variability exists in the literature regarding postoperative immobilization strategies with missing timelines for return to function. Where there is a lack in the evidence base to provide sound postoperative recommendations, a Delphi approach can be successfully used. This study presents consensus-based guidance for postoperative best practice, which can be used as a framework until high-quality evidence-based research is available to form future recommendations. This will be particularly valuable for hand units in the process of newly adopting this surgical technique.

**Take home message**

- Specialist hand surgeons and therapists across the UK and Europe have reached consensus on guidance for postoperative management of thumb carpometacarpal joint replacement (CMCJR).

- The guidance on immediate postoperative practice, rehabilitation, and timescales for return to function can be used to guide postoperative treatment in the absence of robust evidence.

- Dissemination of the consensus-based guidance may help reduce variation and enhance functional outcomes, particularly for hand units in the process of establishing a thumb CMCJR service.

## Data Availability

The data that support the findings for this study are available to other researchers from the corresponding author upon reasonable request.
